# Glioblastoma: Microenvironment and Niche Concept

**DOI:** 10.3390/cancers11010005

**Published:** 2018-12-20

**Authors:** Davide Schiffer, Laura Annovazzi, Cristina Casalone, Cristiano Corona, Marta Mellai

**Affiliations:** 1 Professore Emerito di Neurologia, Università di Torino, Corso Bramante 88/90, 10126 Torino, Italy; davide.schiffer@unito.it; 2 Ex Centro Ricerche/Fondazione Policlinico di Monza, Via P. Micca 29, 13100 Vercelli, Italy; lannov16@gmail.com; 3 Istituto Zooprofilattico Sperimentale del Piemonte, Liguria e Valle d’Aosta, Via Bologna 148, 10154 Torino, Italy; cristiano.corona@izsto.it; 4 Dipartimento di Scienze della Salute, Scuola di Medicina, Università del Piemonte Orientale “A. Avogadro”, Corso Mazzini 18, 28100 Novara, Italy; martamel73@gmail.com; 5 Fondazione Edo ed Elvo Tempia Valenta—Onlus, Via Malta 3, 13900 Biella, Italy

**Keywords:** glioblastoma, microenvironment, niche, pericytes, reactive astrocytes

## Abstract

The niche concept was originally developed to describe the location of normal neural stem cells (NSCs) in the subependymal layer of the sub-ventricular zone. In this paper, its significance has been extended to the location of tumor stem cells in glioblastoma (GB) to discuss the relationship between GB stem cells (GSCs) and endothelial cells (ECs). Their interaction is basically conceived as responsible for tumor growth, invasion and recurrence. Niches are described as the points of utmost expression of the tumor microenvironment (TME), therefore including everything in the tumor except for tumor cells: NSCs, reactive astrocytes, ECs, glioma-associated microglia/macrophages (GAMs), myeloid cells, pericytes, fibroblasts, etc. and all intrinsic and extrinsic signaling pathways. Perivascular (PVNs), perinecrotic (PNNs) and invasive niches were described from the pathological point of view, highlighting the basic significance of the EC/tumor stem cell couple. PNN development was reinterpreted based on the concept that hyperproliferative areas of GB are composed of GSCs/progenitors. TME was depicted in its function as the main regulator of everything that happens in the tumor. A particular emphasis was given to GAMs, pericytes and reactive astrocytes as important elements affecting proliferation, growth, invasion and resistance to therapies of tumor cells.

## 1. Introduction

Glioblastoma (GB) is the most aggressive primary brain tumor in adults accounting for >50% of the tumors of the brain. After surgery, radio- and chemotherapy, survival remains dismal and less than 15 months [1].

Three main properties of the tumor hampers its successful treatment: *(i)* The occurrence of GB stem cells (GSCs); *(ii)* the tumor heterogeneity; *(iii)* the microenvironment and the niches. All these features represent crucial points in the tumor therapy.

The hypothesis of a GSC origin of the tumor is based on the assumption that they represent a rare subset of cells within GB with significant expansion capacity and the ability to generate new tumors [2]. The rest of the tumor is composed of variously differentiated cells with limited progenitor capacity or terminally differentiated non-tumorigenic cells [3]. Therefore, cell heterogeneity and hierarchical organization of GB largely depends on its origin from stem cells or progenitors. Other possible origins of GB are: *(i)* From mature astrocytes that may acquire stemness properties through a dedifferentiation process [4,5]; *(ii)* from neuron glial antigen 2 (NG2) or chondroitin sulphate proteoglyacan 4 (CSPG4)-positive cells, mostly in tumors arising far from the ventricles or with (secondary) *Isocitrate Dehydrogenase* (*IDH*)-mutant GB [6]. Reactive astrocytes may contribute to glioma development, too [7,8]. In fact, they are derived from precursors with a stem-like phenotype [9]. Alternatively, GSCs may represent a sheer functional status [10], depending on the microenvironment regulation [11,12,13,14]. The location and generation of GSCs inside the tumor have long been discussed [3]. They may occur either throughout the tumor [15] or, most probably, in proximity of the central necrosis [16,17,18]. They can be found in the highly proliferative areas of GB close to central necrosis [12,19]. These areas are characterized by high cell and vessel density, high values of proliferation markers, high expression of hypoxia and by the occurrence of circumscribed necrosis; they are in contiguity with the infiltration edges of the tumor.

There is a general agreement that GSCs in the tumor reside in niches that are similar to those hosting normal neural stem cells (NSCs) in the subventricular zone (SVZ) [20]. In these niches, neuroblasts, quiescent NSCs and transit-amplifying cells (A, B and C cells, respectively) occur [21]. They are surrounded by ependymal cells projecting an apical process toward the ventricle. They also develop close to vessels, essential for the stemness maintenance [22]. The main function of the niche in the SVZ is thus to preserve stemness of NSCs [22,23].

The niche concept in malignant gliomas was originally developed to describe the sites where GSCs reside in the tumor and where the tumor microenvironment (TME) exerts its maximum influence. Therefore, for the definition of niche, two conditions must be respected: *(i)* That GSCs do occur and *(ii)* that they have direct contact with endothelial cells (ECs). These conditions are fully realized only in perivascular niches (PVNs) that develop in exchange vessels and not in larger transport vessels with a well-defined layer wall. However, in a broader sense, the term niche also includes perinecrotic niches (PNNs) that do contain GSCs, but not ECs, the occurrence of which precedes necrosis development, being not a reactive phenomenon. On the other hand, GSCs/progenitors in different differentiation stages populate solid proliferative areas of GB, apparently not associated with necrosis or vessels; their differentiation stage is regulated by the TME.

## 2. Pathology of Niches

### 2.1. Perivascular Niches

**Figure 1:**
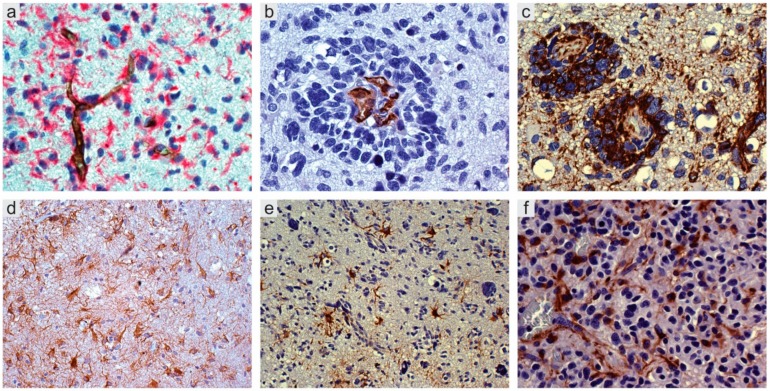
Glioblastoma, IDH wild type. (**a**) CD34+ endothelial cells of arterioles and capillaries in direct contact with Nestin+ tumor cells; double immunostaining with CD34 (DAB) and Nestin (*Fast Red*), original magnification (OM) ×400. (**b**) Vessel co-option. Sleeve of tumor cells around capillaries; CD34, DAB, OM ×400. (**c**) *Id*., with several Nestin+ tumor cells; DAB, OM ×200. (**d**) Mild infiltration. Reactive astrocytes on small vessels; GFAP, DAB, OM ×200; (**e**) More intense infiltration. Reactive astrocytes on vessels; GFAP, DAB, OM ×200; (**f**) High infiltration. Reactive astrocytes with end-feet on small vessels; GFAP, DAB, OM ×400. IDH, isocitrate dehydrogenase; DAB, 3,3′-*Diaminobenzidine*.

A census of the possible cell components, besides tumor cells, would include normal and reactive astrocytes, pericytes, glioma-associated microglia/macrophages (GAMs), myeloid cells, fibroblasts, and, obviously, GSCs and normal NSCs [24,25,26,27]. In their simplest form, PVNs are represented by capillaries or arterioles where ECs are in direct contact with stem cells [28] (Figure 1a). Larger vessels with defined layers, such as transport vessels, cannot function as niches, because they do not allow direct contact between GSCs and ECs. Therefore, not all areas containing vessels and tumor cells would deserve the name niche and are crucial for tumor growth, diffusion, and resistance to therapies [27,28,29,30]. The non-cellular component is given by intrinsic and extrinsic signaling pathways [25].

Niches are mainly found in infiltration and invasion areas of the tumor, where they are called invasive niches [29,31]. In invasion areas, tumor cells infiltrate normal tissue as single cells and grow along the basal lamina of vessels to form the so-called vessel co-option (Figure 1b,c), slipping between vessels and reactive astrocytes [32]; the detachment of their end-feet from vessels contributes to the brain-blood-barrier (BBB) disruption (Figure 1d–f) [33].

**Figure 2:**
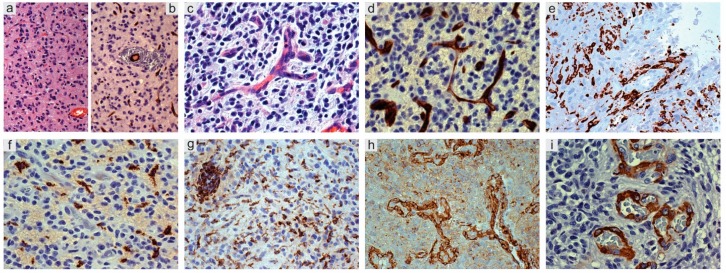
Glioblastoma, IDH wild type. (**a**) Mild infiltration with inital vessel increase; H&E, original magnification (OM) ×200. (**b**) *Id*., CD34+ endothelial cells; DAB, OM ×200. (**c**) More advanced tumor infiltration, small vessel with endothelial proliferation and sprouts; H&E, OM ×400. (**d**) *Id*., CD34+ endothelial cells; DAB, OM ×400. (**e**) Mild infiltration with perivascular macrophages; CD163, DAB, OM ×200. (**f**) Infiltration area with leaked perivascular macrophages; CD163, DAB, OM ×200. (**g**) Ramified microglia in tumor parenchyma and perivascular macrophages; Iba-1, DAB, OM ×200. (**h**) Proliferated tumor vessels with NG2/CSPG4+ endothelial cells and pericytes; DAB, OM ×200. (**i**) Glomeruli with α-SMA pericytes; DAB, OM ×200. IDH, isocitrate dehydrogenase; H&E, hematoxylin and eosin; DAB, DAB, 3,3′-*Diaminobenzidine*.

Reactive astrocytes produce angiopoietins 1 (Ang-1) and 2 (Ang-2) and vascular endothelial growth factor (VEGF) [34,35,36]. Nestin+ and Sox2+ tumor cells representing the neoplastic counterpart of normal progenitor cells. They trigger pericyte dissociation, matrix and basal lamina degradation, vessel dilation, leakiness and extracellular deposition of fibrin, to form the so-called “mother vessels” or chronic hyperplasia [37]. In the absence of inhibition from pericytes, ECs proliferate (Figure 2a,b); a switch from an avascular to a vascular state follows and sprouts are formed through EC proliferation (Figure 2c,d). BBB undergoes disruption with leak of macrophages from the vessels (Figure 2e–g). Pericytes, recruited by platelet-derived growth factor receptor β (PDGFRβ) [38], dissociate and ECs proliferate to form new channels that cover with an increased number of pericytes (Figure 2h,i). Whether they are venules or arterioles or neo-formed tumor vessels that do not correspond to any type of normal vessels is difficult to demonstrate. Hypoxia obviously occurs, as everywhere in GB. Glomeruli appear later, surrounded by macrophages and reactive astrocytes. In gliomas, glomeruli formation during angiogenesis takes place as in normal embryos, with the difference that, in the tumor, it is dysregulated and bumpy structures are built that do not contribute to the supply of nutrients and oxygen to the tumor [39].

Angiogenesis is not the only possibility the vasculature has to expand, since vasculogenesis and trans-differentiation of tumor cells into ECs may occur as well [35,40,41].

### 2.2. Perinecrotic Niches

**Figure 3:**
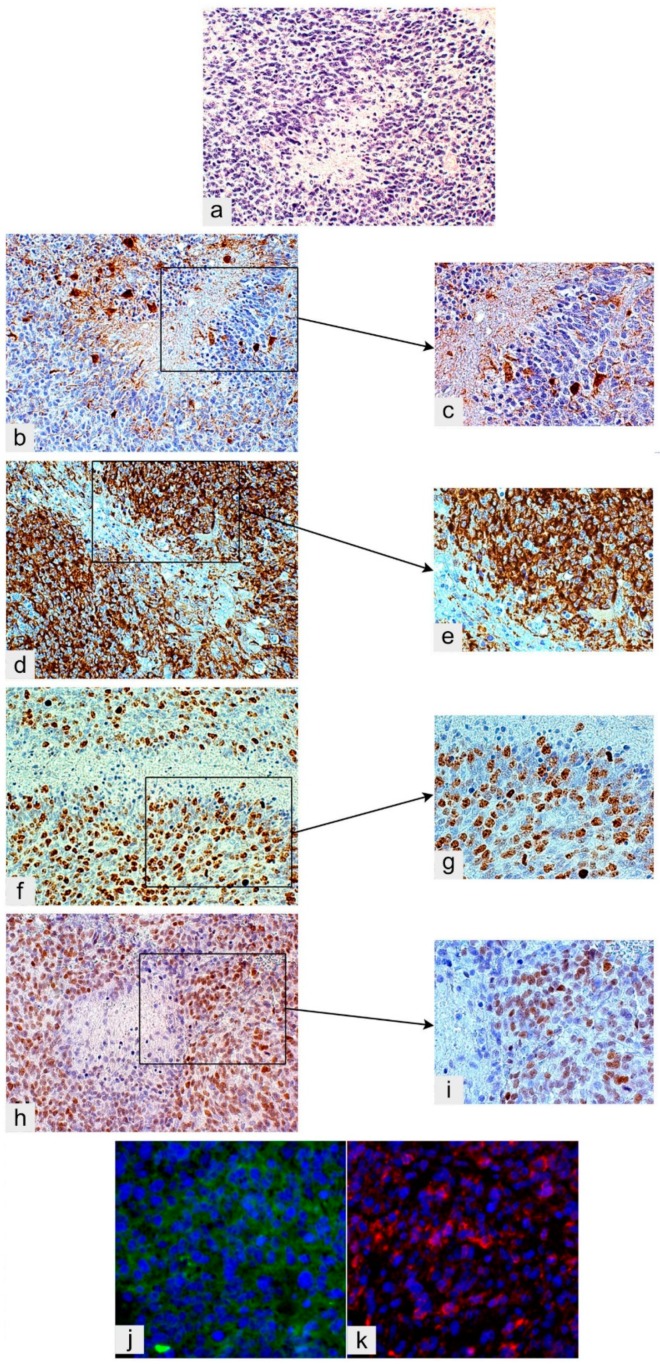
Glioblastoma, IDH wild type. (**a**) Circumscribed necrosis in a hyperproliferative area; H&E, original magnification (OM) ×200. The hyperproliferative zone bordering necrosis is almost GFAP-negative; DAB, OM ×200 (**b**) and ×400 (**c**). The same area is highly Nestin-positive; DAB, OM ×200 (**d**) and ×400 (**e**). The same area shows a high Ki-67/MIB-1 labeling index; DAB, OM ×200 (**f**) and ×400 (**g**). The same area is highly Sox2-positive; DAB, OM ×200 (**h**) and ×400 (**i**). The same area is positive for Musashi-1, cryostat section, immunofluorescence (green) (**j**) and highly CD133-positive, cryostat sections, immunofluorescence (red), both OM ×400 (**k**). IDH, isocitrate dehydrogenase; H&E, hematoxylin and eosin; DAB, DAB, 3,3′-*Diaminobenzidine* [49].

Classically, circumscribed necrosis has been interpreted as due to a vessel occlusion or an intravascular thrombosis [42]. Perinecrotic pseudopalisades have been considered as due to tumor cells fleeing necrosis [43]. GSCs would be induced by hypoxia and hypoxia-inducible factor 1 (HIF-1) and 2 (HIF-2) [35]. Alternatively, or additionally, GSCs/progenitors are believed to regularly populate hyperproliferative areas of GB near central necrosis with high cell and small vessel density, several mitoses and a high Ki-67/MIB-1 labeling index [3]. These areas are regulated by the microenvironment and are recognizable because of their Nestin, Sox2 and CD133 positivity (Figure 3a–k). In these areas, circumscribed necrosis develops as the result of the imbalance between the high proliferation rate of tumor cells and the low one of ECs [44,45]. Indeed, circumscribed necroses are always found in avascular areas of hyperproliferative districts (Figure 3a), close to central necrosis of GB. The cell population expressing Nestin, Sox2 and other stemness markers, including CD133, remains to border the necrosis as remnants of GSCs/progenitors that populated the area and escaped necrosis [12,13] (Figure 3d,e,h–k).

A recent paper took into consideration all niche types described in the literature (perivascular, hypoxic, immune, extracellular matrix niches, etc.) concluding that they are not distinct from one another but they are parts of a single GSC niche, according to the hypoxic periarteriolar niche model [46,47] in which cathepsin K would play a functional role [48]. Roughly, they correspond to the one described as prototype of the PVN.

## 3. Tumor Microenvironment (TME)

TME represents the non-cancerous cells inside the tumor, including normal and reactive astrocytes, GSCs, fibroblasts, immune cells, microglia/macrophages, ECs and vascular pericytes. It also includes proteins and non-protein biomolecules (polysaccharides, hormones, nitric oxide (NO), etc.) produced by all cell types within the TME to support the tumor growth. TME can be mainly detected and demonstrated in niches, but is supposed to regulate everything in the tumor and in the tissue around the tumor or in the brain adjacent to the tumor. GSCs, for instance, mainly occur in niches, but they can also be found in proliferative areas of GB as well [13,50,51], even conceived as being of hypoxic origin. As a matter of fact, hypoxia occurs in the whole tumor, distributed with variable intensity. In necrotic foci, it assumes the typical phenotype of necrosis [52,53], but it may occur in a spot-like manner, with minor intensity and with a not yet modified phenotype. Therefore, besides central and circumscribed necrosis, isolated tumor cells may undergo death as phenotypic hypoxia translation because of individual tumor cell responses to a range of oxygen tension [29]. Other examples are available in the brain tumor pathology. Single apoptotic cells may occur in proliferative areas, due to both the intrinsic, transcriptional pathway of apoptosis associated with duplication, and to the extrinsic pathway associated with a phenotypically subliminal necrosis [3].

**Figure 4:**
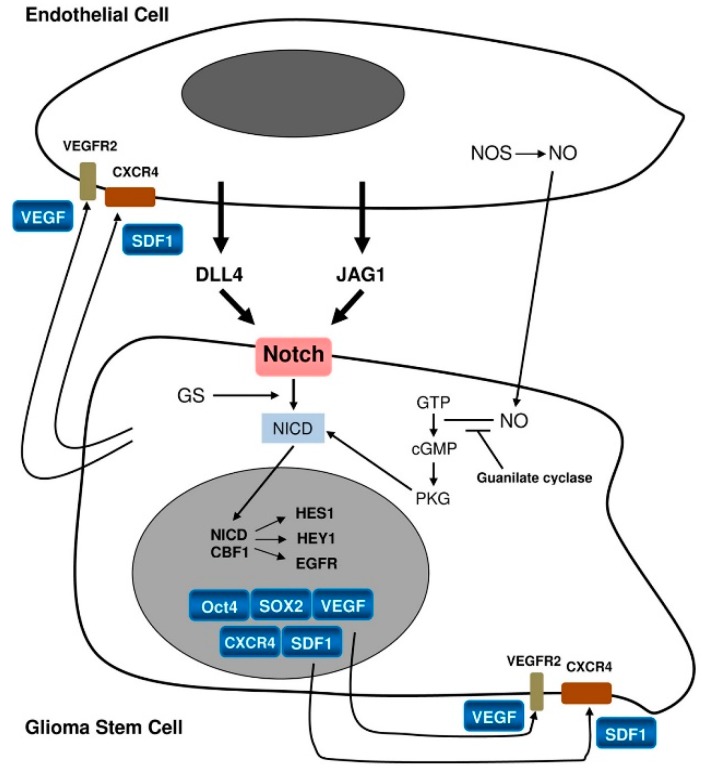
Relationship between a stem cell/progenitor and an endothelial cell [13].

In PVNs, the most important signaling is supposed to occur between GSCs/progenitors and ECs [3] (Figure 4).

The stemness status of GSCs/progenitors is maintained by ECs via pathways such as NO, cyclic guanosine monophosphate (cGMP) [46] and Notch activation [13,25,54]. Notch-1 and Notch-2 are expressed on GSCs whereas their ligands, Delta-like ligand 4 (DLL4) and Jagged 1 (JAG1), are expressed on the ECs [55]. GSCs/progenitors would promote EC proliferation, eliciting angiogenesis through VEGF, and hosting the bone marrow-derived endothelial precursor cells (EPCs) at the tumor. After activation through its ligands Notch also leads to the final activation of target genes such as *HES1* and *HEY1* [51,56]. This has been confirmed by the blockade of Notch by γ-secretase inhibition that reduces the expression of stemness antigens such as Nestin, CD133 and Bmi-1. It also inhibits in vitro human GB-derived neurosphere formation and xenografts [57] promoting their differentiation into blood vessels inserted into the pre-existing vasculature [27].

Another efficient factor is hypoxia, considered to be a hallmark of GB [53,58,59,60,61] that activates pro-angiogenic factors, such as Ang-1/2, transforming growth factor β (TGF-β), PDGF-BB/PDGFR and VEGF/VEGFR through HIF-1/2 [62]. Therefore, hypoxia triggers multiple signaling pathways that affect GSCs self-renewal, proliferation, cell invasion and survival [63]. In addition, it also influences therapeutic resistance of GB and enhances genetic instability of tumor cells. The low oxygen content in the tumor tissue attenuates the expression of *DNA Mismatch Repair (MMR)* genes and inhibits free radicals generated from radiation treatment thus impeding therapeutic efficacy. The *Multi-Drug Resistance Gene 1 (MDR1/ABCB1)* encoding for P-glycoprotein (P-gp) is activated in response to hypoxia [64]. A complicated series of spatially heterogeneous tissue events follows hypoxia in GB [including energetic metabolism (29)] promoting the malignant phenotype and tumor heterogeneity [65].

**Figure 5:**
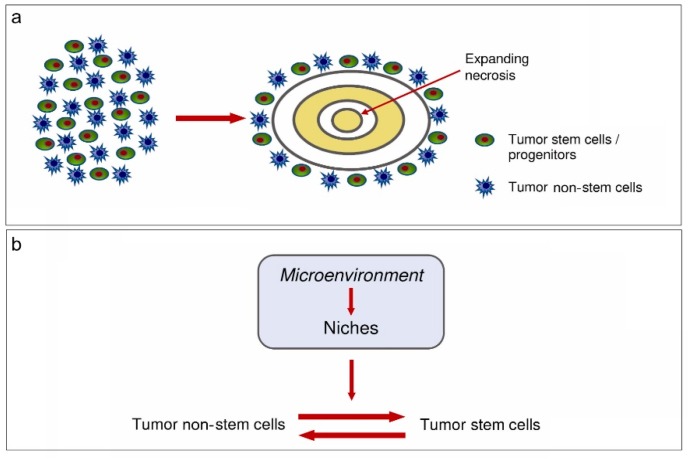
(**a**) Development of circumscribed necrosis. (**b**) Equilibrium between tumor non-stem cells and tumor stem cells [66].

Finally, TME may control the regulation of the equilibrium between tumor stem and non-stem cells (Figure 5).

## 4. Glioma-Associated Microglia/Macrophages (GAMs)—Inflammatory Microenvironment

GAMs cannot be interpreted using the same criteria as for macrophages in other pathological conditions. A fundamental distinction is made between: *(i)* Resident microglia-derived cells (i.e., the so-called reactive microglia with a typical histological appearance) and *(ii)* blood-borne macrophages. The described dichotomy is given with a certain degree of approximation as, often, the distinction between the two cell types is not so sharp. However, different types of myeloid cells occur. It is worth considering that both resident microglia and blood-borne monocytes derive from the yolk sac in different times during embryonic development, the former earlier and directly, and the latter later and through the bone marrow.

The great amount of GAMs, almost equal to the number of tumor cells, raises many still unanswered questions. For instance, GAMs are mainly identified as reactive ramified microglia in low-grade gliomas (LGGs) and as blood-borne monocytes in high-grade gliomas (HGGs) (Figure 2e–g). Since microglia/macrophages and other myeloid cells are strictly connected with the immunological features of gliomas, there is wide literature on the subject suggesting various therapeutic strategies. Basically, there is compelling evidence that GAMs favor tumor progression [67,68,69,70,71,72,73,74,75,76,77,78,79], but uncertainties concerning the M1/M2 polarization and the extent of phagocytosis still exist. Very likely, the dilemma whether they are “friends or foes” [80] has not been completely solved because of some demonstrations on the “good” role of GAMs. Scavenger receptors and phagocytosis seem to be completely lacking; however, Fc-γ receptor expression occurs in the solid tumor and, at a lesser extent, in the peritumoral tissue [81,82].

Monocytes [83], tumor-associated neutrophils (TANs) [84] and myeloid-derived suppressor cells (MDSCs) [85] are commonly found within the TME [70,86,87,88]. An intense signaling exchange takes place among MDSCs, ECs, macrophages, tumor cells and reactive cells [29]. In addition, the influence of chemokines and their receptors must be considered. The most studied signal axes include CXCL12 (SDF-1)-CXCR4, CXCL2-CXCR2, CCL2-CCR2, CX3CL1-CX3CR1, but the problem is far from being completely clarified. The first interaction occurs between macrophages and GSCs [83,89] with the latter activating M2 anti-inflammatory macrophages and, conversely, being maintained in stemness through CXCL12 (SDF-1)-CXCR4 axis. GSCs secrete periostin that recruits M2 tumor-associated macrophages (TAMs) and promotes glioma growth through intergin α_v_β3 [90]. Moreover, TGF-β, released from TAMs, induces matrix metalloproteinase 2 (MMP-2) and 9 (MMP-9) expression from the tumor to enhance GSC invasion [91,92,93,94]. On the other hand, TGF-β, shed from GSCs, promotes the polarization of microglia/macrophages into the M2 immunosuppressive phenotype enhancing the capacity of TAMs to inhibit T cell proliferation, thereby promoting tumor progression [25,95]. MDSCs mediate immune suppression and support glioma growth, also interacting with GSCs [96], mainly by immunosuppressing monocytes and other T cell populations [29].

GB can be classified into Proneural (PN), Neural (N), Mesenchymal (MES) and Classical (CL) subtypes, each with its own GSC content [97]. MES GSCs show a preferential activation of the Notch signaling pathway and PDGF receptor, whereas activation of the nuclear factor-κB (NF-κB) pathway and glycolysis-mediated metabolism pathway prevail in PN GSCs. Radiation therapy may induce in GSCs a cellular transformation resembling the epithelial-mesenchymal transition (EMT), called proneural-mesenchymal-transition (PMT) [98]. Triggering PMT GSCs are maintained, and in this step crucial is osteopontin (OPN) that, secreted by immune cells, promotes GSCs phenotype by activating CD44 [99]. A complicated mechanism involves PN and MES GB expression subtypes, PMT, CD44, tumor necrosis factor α (TNF-α), but how GSCs, ECs and TAMs interact has not yet been completely understood. Several studies on microglia/macrophages in gliomas focused on improving patient survival [70,100,101]; some of them, mainly in recent times, concerned the use of dendritic cells (DCs). DCs are granular lymphocytes with cell surface markers: major histocompatibility complex (MHC) class I molecules, MHC class II molecules and CD86, all of which can help to identify DCs from other myeloid lineage cells [102]. They recognize and bind antigens in their immature state and then migrate to lymphoid organs where they present processed peptides to T cells in the context of MHC I or II molecules [103,104] inducing tumor antigen-specific immune responses. Additionally, DCs display various features in the immune system that balance the complex system of inflammatory and inhibitory immune reactions in the TME [105]. Several studies have been designed with a therapeutic task [106,107,108,109].

## 5. Pericytes

There is a cross-talk among vascular pericytes and the other components of the TME, mainly ECs and GSCs. Their interactions during tumor angiogenesis have been widely discussed. Basically, aberrations in pericyte-EC signaling networks have been regarded as contributing to tumor angiogenesis [110]. Pericytes promote vascular maturation, express PDGFRβ, α-smooth muscle Actin (α-SMA), Desmin and NG2/CSPG4 (Figure 2h,i). Pericytes originate from mesoderm-derived mesenchymal stem cells (MSCs) or from neuroectoderm-derived neural crest cells. They are an essential element of the neurovascular unit and participate in the function of BBB. Their reciprocal signaling with ECs, mainly through PDGFRβ and CXCL12 (SDF-1)-CXCR4, TGF-β and Ang-1 has been widely discussed [111]. Pericytes may derive from GSCs undergoing mesenchymal differentiation and support vessel function and tumor growth. GSCs are recruited toward ECs via the CXCL12 (SDF-1)-CXCR4 axis and induced to become pericytes predominantly by TGF-β. Thus, GSCs contribute to vascular pericytes that may actively remodel PVNs [112].

NG2/CSPG4 promotes tumor growth as a component of both tumor and stromal cells; it is expressed by other cell types, mainly oligodendrocyte precursor cells (OPCs). In myeloid-specific and pericyte-specific NG2/CSPG4 null mice, a reduced growth of the tumor was observed. The loss of pericyte-EC interactions reduces the formation of endothelial junctions, assembly of the basal lamina and reduces macrophage recruitment [113]. MSCs injected into brain tumors in mouse models resulted in close associations with the tumor vasculature, also with up-regulation of the expression of pericyte markers [25]. Through the NG2/CSPG4 knockdown in pericytes by small interfering RNA (siRNA) transfection, 60% reduction of β1 integrin activation and 40% of FAK phosphorylation occur with a concomitant decrease of pericyte proliferation and migration [114]. The NG2/CSPG4 ectodomain, shed from pericytes due to a proteolytic cleavage, may recruit at a distance ECs to sites of angiogenesis and may activate β1 integrin on ECs.

In the neo-angiogenesis of GB, pericytes start increasing together with the disruption of BBB becoming a good marker of neo-vascularization [31].

## 6. Reactive Astrocytes

Reactive astrocytes are a constant phenomenon associated with gliomas [115]. They can surround the tumor or can be located inside. Outside the tumor, they can be found in early or in mature stages. In the first case, they are GFAP+ and Nestin+, regularly distributed with round cytoplasms and short processes, often in mitosis. In the second case, they are more regularly distributed, mainly GFAP+ and with several and long processes. Inside the tumor, they can be observed in continuity with the peritumoral gliosis or entrapped in the advancing tumor with a large, gemistocytic type cytoplasm. They are often distributed around vessels, or they may form areas with a dense GFAP+ fibrillary net. Reactive astrocytes can also be located in highly proliferative areas, around circumscribed necrosis. This means that they may persist for a long time inside the tumor, often in the form of round, GFAP+ cells. The tumor growth speed plays an important role in the reactive astrocyte morphology; in fact, slow growing tumors may include mature astrocytes. The distinction between reactive astrocytes and tumor cells is not easy [116]: the finding of a GFAP+ cell in mitosis does not rule out the possibility that it could be a reactive astrocyte. However, their histological aspect and distribution has been known for a long time and most GFAP positivity of cells in (primary) *IDH*-wild type GB, must be ascribed to entrapped reactive astrocytes [39]. From the functional point of view, peritumoral gliosis cannot be compared with gliosis in other pathological conditions and, for this reason, it must deserve a different interpretation. Notably, reactive astrocytes from tumor infiltration areas send end-feet to arterioles and capillaries, from which they are detached by infiltrating tumor cells, thus contributing to BBB disruption. In addition, reactive astrocytes play a major role in the TME.

There are recent and exhaustive reviews on the subject [117]. Currently, the general opinion is that reactive astrocytes favor invasion and progression of gliomas exerting a chemoprotection and an immune protection of tumor cells. Reactive astrocytes interact with glioma cells and facilitate the progression, aggression and survival of tumors by releasing different cytokines. This interaction is further promoted through ion channels and ion transporters that enhance the migratory capability and invasiveness of tumor cells by modifying H^+^ and Ca^2+^ concentrations and stimulating cell volume changes [115].

Several mechanisms involved in the cross-talk between reactive astrocytes and gliomas favor their proliferation, invasion and resistance to radio- and chemotherapy:

*(i)* Expression of MMP-2, which favors infiltration and secretes CXCL12 (SDF-1) for proliferation and migration [118];

*(ii)* synergistic relationship with tumor cells concerning the p53 function between apoptosis and proliferation [119];

*(iii)* regulation through NF-κB activated by receptor activator of NF-κB ligand (RANKL) and lipopolysaccharides (LPS) that decreases IkBα [120,121];

*(iv)* the gap junction channel protein 43 (Cx43) that confers resistance to glioma cells and prevents apoptosis [122];

*(v)* indirect cross-talk via chemokines (interleukin 6, IL-6), TGF-β, insulin-like growth factor 1 (IGF-1), monocyte chemotactic protein 4 (MCP-4), interleukin 19 (IL-19), VEGF and leukemia inhibitory factor (LIF), promoting tumor cell invasion [123,124];

*(vi)* microRNAs [125,126], oncogene astrocyte elevated gene-1 (AEG-1), which is associated with poor survival of gliomas [127] and acts modulating PI3K/Akt, NF-kB, MMP-2 and MMP-9 [128,129] the inhibition of which induces apoptosis [130];

*(vii)* L-Glutamin [117].

In a murine glioma resection and recurrence model, surgical resection has been showed to alter the reactive astrocyte component of the peritumoral microenvironment and injured astrocytes to induce in vitro alterations of transcriptome and secretome that significantly influence tumor biology. This may be important for therapies [131].

All available speculations on the significance of reactive astrocytes are based on in vitro experiments or on the demonstration that certain pathways play a role in tumor progression. It is possible that these pathways belong to the tumor cells themselves. Moreover, there is no direct demonstration of a negative influence of reactive astrocytes on survival in human gliomas. The possibility that reactive astrogliosis opposes tumor invasion, without success, cannot be completely ruled out. Another unanswered question is whether entrapped reactive astrocytes in the advancing tumor may transform into tumor cells.

## 7. Conclusions

The great amount of contributions on radio- and chemotherapy did not substantially modify survival of GB patients. Studies on cell death-based treatments continue [132] and new approaches are suggested [133], but more recently, studies on immunity of GB have appeared in the literature and special attention is being paid to vaccines, cytokines, DCs, gene therapy and viruses [134,135,136]. This seems to be a possible path to advantageous novelties.
